# Risk Assessment of Clinical Reactions to Legumes in Peanut-Allergic Children

**DOI:** 10.1097/WOX.0b013e3181865f83

**Published:** 2008-10-15

**Authors:** Louise Bjerremann Jensen, Milene Andersen, Per Stahl Skov, Lars K Poulsen, Carsten Bindslev-Jensen

**Affiliations:** 1 Department of Agricultural Sciences, Faculty of Life Sciences, University of Copenhagen, Copenhagen, Denmark; 2 Laboratory of Medical Allergology, Allergy Clinic, National University Hospital, Copenhagen, Denmark; 3 Allergy Centre, Odense University Hospital, Sdr. Boulevard 29, DK-5000 Odense, Copenhagen, Denmark; 4 RefLab ApS, National University Hospital, Copenhagen, Denmark

**Keywords:** cross-reactivity, histamine release, lupine, oral food challenge, pea, peanut allergy, skin prick test, soy, specific IgE

## Abstract

Peanut-allergic children might be at risk for reactions to other legumes. However, it is not always possible to perform multiple oral food challenges in children. On the basis of patient case history, in vitro diagnostic tests, and eventually food challenges, we aimed at developing an algorithm for risk assessment of possible clinical reactions to other legumes (soybean, lupine, fresh, and blanched green pea). Seventy-five consecutive patients with a positive oral food challenge to peanut were included in the study. All tests were run as part of the routine allergy examination. A high proportion of patients and/or caretakers refused the administered legume oral food challenges. Obtained diagnoses from histamine release did not correlate significantly to the outcome of the algorithm. Interestingly, threshold from peanut challenges did not correlate with the risk assessment.

The algorithm presented in this study can be used when advising peanut-allergic children and their caretakers about what other legumes to avoid in the diet.

## 

Due to the severity of peanut-allergic reactions, it is important to have specific and standardized methods for allergy diagnosis. The double-blind placebo-controlled food challenge is considered to be the gold standard for food allergy diagnosis. In children below 3 years, it is accepted to perform open food challenges [1]. The risk associated with an oral food challenge is outweighed by the benefits provided to patients that are shown to be less sensitive (or even tolerant) to the food over time [2,3]. Moreover, even the most sensitive patients and/or their caretakers benefit from a realistic knowledge of their personal threshold.

Cross-reactivity is observed when preexisting immunoglobulin E (IgE) antibodies recognize similar epitopes on other proteins than the protein they were originally sensitized to,[4]and several studies on legume cross-reactivity have been published. Magni et al[5]investigated the degree of in vitro serologic cross-reactivity between peanut allergens and allergens from soybean and lupine seeds by Western blotting and 2D gel electrophoresis. They found that IgE antibodies specific for Ara h 3 peanut allergens (an 11S globulin) also recognized soybean and lupine 11S globulin basic subunits [5]. Wensing et al[6]reported that patients sensitized to pea exhibited clinically relevant cross-reactivity to peanut where the protein responsible for the cross-reaction was vicilin (Ara h 1 in peanut) [6].

A serological cross-reactivity is, however, not synonymous with allergic reactions in the patients. Bernhisel-Broadbent and Sampson[7]and Bernhisel-Broadbent et al[8]found in a pediatric population an extensive serologic cross-reactivity between legume species that was not reflected clinically. On the one hand, it is argued that related allergens can cause unexpected clinical reactions and, on the other hand, that these structures can mimic sensitization without clinical manifestation, which may be a concern in the diagnosis of patients[9].

Although oral food challenges would be ideal to establish clinical reactivities to other legumes, it is clearly not feasible to perform 4 to 8 additional food challenges in peanut-allergic children in the daily routine.

Because peanut-allergic children may be at risk for reactions to legumes but cannot always be challenged with all suspected foods, we aimed at developing an algorithm for risk assessment of possible clinical reactions to other legumes (soybean, lupine, fresh, and blanched green pea). On the basis of case history, in vitro diagnostic tests, and, when accepted by the patient, food challenge, the child was classified to a high- or a low-risk group. Furthermore, we compared the threshold for peanut challenge and tested the ability of histamine release (HR) as independent diagnostic tools to discriminate between high- and low-risk patients.

## Materials and methods

### Patients

The inclusion criterion for the study was a peanut allergy confirmed by oral food challenge. Seventy-five consecutive patients were included in the study: 46 males (61%) and 29 females (39%). All patients attended the Allergy Centre at Odense University Hospital, and all tests were run as a part of the routine allergy examination in the period from November 2000 to November 2005.

All patients but 1 were children or adolescents. The age of the patients at the time of the positive oral food challenge was between 7 months and 42 years, the median age being 5 years and 9 months. One patient was not subjected to an oral food challenge with peanut due to a clear-cut case history of peanut allergy but was still included in the study as peanut challenge positive according to the European Academy of Allergology and Clinical Immunology guidelines [1].

Titrated peanut challenges were performed as described by Taylor et al,[10]and the content of peanut in the challenge meal inducing subjective and/or objective symptoms was determined. Also, the cumulative dose amount of peanut was recorded. The dose yielding the first mild objective symptoms corresponds to the lowest observed adverse effect level as reported by Taylor et al.[11]Detailed description of the threshold doses obtained from the peanut challenges will be published elsewhere.

The investigations applied were routine procedures, and therefore the local ethics board stated that formal approval was unnecessary. All testing was only carried out in accordance with the parents and patients wishes.

### Legume-Specific Risk Assessment

After diagnosis of peanut allergy based on the positive challenge outcome, it was investigated whether the patients reacted to other legumes. The patients and/or their caretakers were asked about observed reactions to soybean, lupine, fresh green pea, and blanched green pea, which all belong to the botanical legume family. The answers obtained were grouped in 3 categories: "case history positive or avoids," which included the patients that experienced symptoms upon ingestion and the patients that avoided the food to prevent the induction of symptoms from possible cross-reactions; "case history negative and eats regularly," which included the patients that ate the food without experiencing symptoms; and "case history unknown," which included patients with no known case history. The patients with a positive case history or that avoided the foods were administered a food challenge, but they all refused due to fear of eliciting symptoms during challenge. These patients were categorized as having a high risk of experiencing a reaction after legume consumption. The patients with an unknown case history were subjected to skin prick test (SPT) and/or measurement of specific IgE (sIgE). If 1 or both results came out positive, the patients were administered a food challenge. If the challenge was refused or the outcome of the challenge was positive, the patients were grouped in the "high-risk" group. A negative challenge outcome grouped the patients in the "low-risk" group. The patients with negative SPTs and sIgE diagnoses were categorized as "low-risk" patients based on the high negative predictive value of SPT and sIgE diagnoses [7,12]. Finally, the patients that had a negative case history and ate the legume foods regularly were grouped in the "low-risk" group.

**Figure 1:**
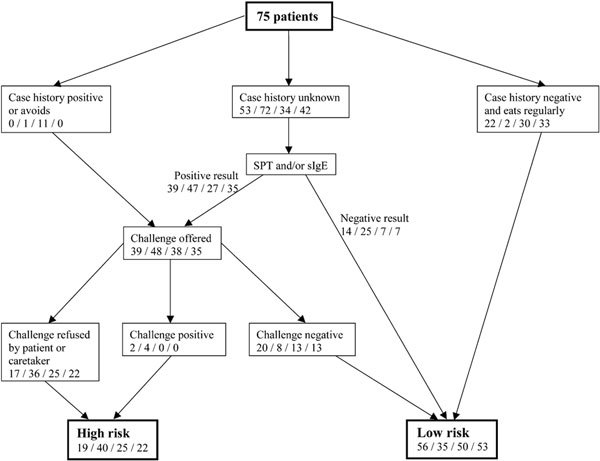
**Flow sheet of the algorithm**. The numbers in each box correspond to the number of patients in the risk assessment of possible reactions to soybean/lupine/fresh green pea/blanched green pea, respectively. Eventually, the patients are divided into high- or low-risk groups. Pea challenges were only performed with fresh green pea. The outcome of the fresh pea challenge was used to classify the patients as either high or low risk for both fresh and blanched green pea.

The flow sheet in Figure 1 depicts the algorithm by which the patients were classified into 2 groups, reflecting either a high risk or a low risk of possible reaction to the legumes. The outcome of this risk assessment was used in the guidance of the patients and/or caretakers.

### Legumes

Commercially available products of peanut (*Arachis hypogaea*), soybean (*Glycine max*), lupine (*Lupinus angustifolius*), and green pea (*Pisum sativum*) bought locally were tested in the study because they are common legumes in the diet in Denmark. Both fresh and blanched green peas were tested, where blanched green peas were peas that were briefly blanched in the manufacturing process before they were frozen and sold.

### Skin Prick Test

Skin prick test was performed with peanut, soybean, lupine, fresh green pea, and blanched green pea by the prick-prick method. Also, a negative (saline) and positive control (histamine; ALK-Abelló, Hørsholm, Denmark) was included. The criterion for positive result of the test was a wheal with a diameter of 3 mm or greater, a negative saline control result, and a positive histamine control result.

### Measurement of sIgE

Serum samples were analyzed for IgE specific for peanut, soybean, lupine, and green pea using the Phadia ImmunoCAPTM system based on a fluoroenzyme immunoassay (Phadia, Uppsala, Sweden). The cutoff for a positive diagnosis was 0.35 kUA/L.

### Direct HR

Patients were tested in basophil HR by direct stimulation of patient basophils. Heparinized blood from the patients was washed with piperazine-*N*-*N*'-*bis*-2-ethanesulfonic acid (PIPES), pH 7.4 (3.02 g/L PIPES, 19.05 g/L sodium acetate, 0.49 g/L potassium acetate, 1.0 M Tris buffer, 0.088 g/L sodium chloride, 0.224 g/L magnesium chloride; RefLab ApS, Copenhagen, Denmark). Supernatant was discarded, and cells were resuspended in PIPES with interleukin-3 and stimulated with legumes in 6 different concentrations. The legumes used were peanut, soybean, lupine, fresh green pea, and blanched green pea. Legume extracts were performed as follows: 1 g of legume in 10 mL PIPES was grinded in a Stomacher 80 (Seward Medical Limited, London, UK) for 60 seconds at high speed with 10 mL. The sample was centrifuged (3000 × *g*, 10 minutes, 5°C), and the supernatant was saved as stock solution. The stock solution was diluted 1:10, and this dilution was used as the strongest dilution. Five subsequent 3.5-fold dilutions were prepared. The legume dilutions (25 μl) were applied to the glass microfiber plates together with 25 μL patient blood sample containing 2 ng/mL interleukin-3.

The histamine was measured spectrofluorometrically according to the glass microfiber method HR-Test at RefLab ApS described by Stahl Skov et al.[13]An HR of 10 ng/mL or greater was considered a positive test result. Positive results were classified in HR classes 1, 2, and 3, where patients with positive reactions to 1 or 2 dilutions were classified as HR class 1, patients reacting positive to 3 or 4 dilutions were classified as HR class 2, and patients reacting positive to 5 or all 6 dilutions were classified as HR class 3 [14].

Histamine release was not a part of the allergy diagnostic workup and was only performed if there was an indication of a reaction. Therefore, HR data were not included in the algorithm.

### Statistics

Specific IgE results were compared with case history outcomes by *t *test. Obtained HR classes were compared with the classification of patients into high- and low-risk groups by *χ*^2 ^test. Threshold levels from peanut challenges were compared with the classification of patients into high- and low-risk groups by *t *test.

## Results

All 75 patients in the study had a clinically confirmed peanut allergy based on the European Academy of Allergology and Clinical Immunology guidelines [1].

Table 1 shows frequencies of positive results obtained with the 3 different diagnostic tests: SPT, HR, and sIgE. The diagnostic tests SPT and HR produced most positive results for lupine and fresh green pea, followed by soybean and blanched green pea. The measurements of sIgE gave most positive results for soybean followed by pea and lupine.

**Table 1:** Frequencies of Positive Diagnostic Tests in Peanut-Allergic Patients

Legume	SPT	HR	sIgE
Peanut	97% (68/70)	92% (45/49)	97% (69/71)
Soybean	28% (17/60)	19% (9/47)	58% (37/64)
Lupine	45% (23/51)	80% (43/54)	47% (22/47)
Fresh green pea	41% (17/41)	91% (39/43)	48% (23/48)
Blanched green pea	16% (6/38)	5% (2/43)	

Values in parentheses are the number of patients with positive outcome of the tests divided by the number of patients having the tests performed.

Pairwise comparisons between the 3 diagnostic tests were performed (Table 2). In 4 of 5, the concordance was lowest between HR and sIgE.

**Table 2:** Concordances for Pairwise Comparison of the Diagnostic Tests: SPT, HR, and sIgE

Legume	SPT vs HR, %	SPT vs IgE, %	HR vs IgE, %
Peanut	88	97	90
Soybean	80	70	57
Lupine	67	67	57
Fresh green pea	53	63	52
Blanched green pea	79	63	57

The flow sheet depicting the developed algorithm for risk assessment of the patient's potential reaction to other legumes is depicted in Figure 1. A high fraction of the patients had an unknown case history to the legumes (71% for soybean, 96% for lupine, 45% for fresh green pea, and 56% for blanched green pea). The patients in this group with either a positive SPT and/or sIgE were administered an oral food challenge.

A high proportion of patients and/or caretakers refused the oral food challenge (44% for soybean, 75% for lupine, 66% for fresh green pea, and 65% for blanched green pea). The positive case history or the positive result of the in vitro diagnosis may explain these high proportions of patients refusing challenge.

The fraction of the patients designated to the high-risk group was 25% for soybean, 53% for lupine, 33% for fresh green pea, and 29% for blanched green pea.

Table 3 depicts the means of SPT and HR, as well as the median of sIgE measurements, where the patients were grouped in positive, negative, and unknown case history. Significant differences (*P *< 0.05) were observed for sIgE results for fresh green pea between patients with positive and negative case history (*P *= 0.007) and for sIgE results for blanched green pea between patients with negative and unknown case history. Generally, the mean for SPT and HR and the median for sIgE were lowest for patients with negative case history (*P *= 0.047).

**Table 3:** Mean SPT, Mean HR Class, and Median sIgE for Patients Grouped Into Positive, Negative, or Unknown Case History (Number of Patients in Brackets)

		Positive Case History	Negative Case History	Unknown Case History
Soybean	SPT		1 (21)	2.5 (41)
	HR		0.1 (19)	0.5 (28)
	sIgE		0.3 (21)	0.7 (45)
				
Lupine	SPT	--(0)	4 (2)	2.5 (54)
	HR	--(0)	0 (1)	1.3 (25)
	sIgE	--(0)	--(0)	0.3 (47)
				
Fresh green pea	SPT	2.7 (7)	1.5 (23)	2.8 (30)
	HR	1.3 (6)	1.0 (24)	1.2 (25)
	sIgE	1 (6)	0.3 (20)	0.4 (25)
				
Blanched green pea	SPT		1.2 (27)	1.9 (32)
	HR		0.2 (29)	0.2 (26)
	sIgE		0.3 (28)	0.9 (23)

**Figure 2:**
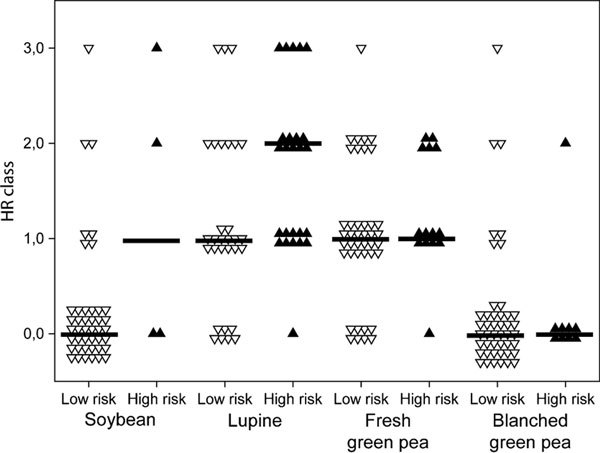
**Comparison between high- and low-risk classification and HR class for the soybean, lupine, fresh green pea, and blanched green pea**. Black lines represent the median.

The outcome of the HR diagnostic test was compared with the classification of patients into high- and low-risk groups. Figure 2 shows the distribution of the HR classes between high- and low-risk groups. There is a tendency for soybean and lupine that HR classes are higher for patients in the high-risk groups; however, this was not significant. Figure 2 indicates that the HR test was too sensitive for fresh green pea.

Peanut HR classes were also compared with the high- and low-risk classification. No difference was observed, partly explainable by the fact that 39 of 49 patients were HR class 3 on which the HR analyses was performed (data not shown).

**Figure 3:**
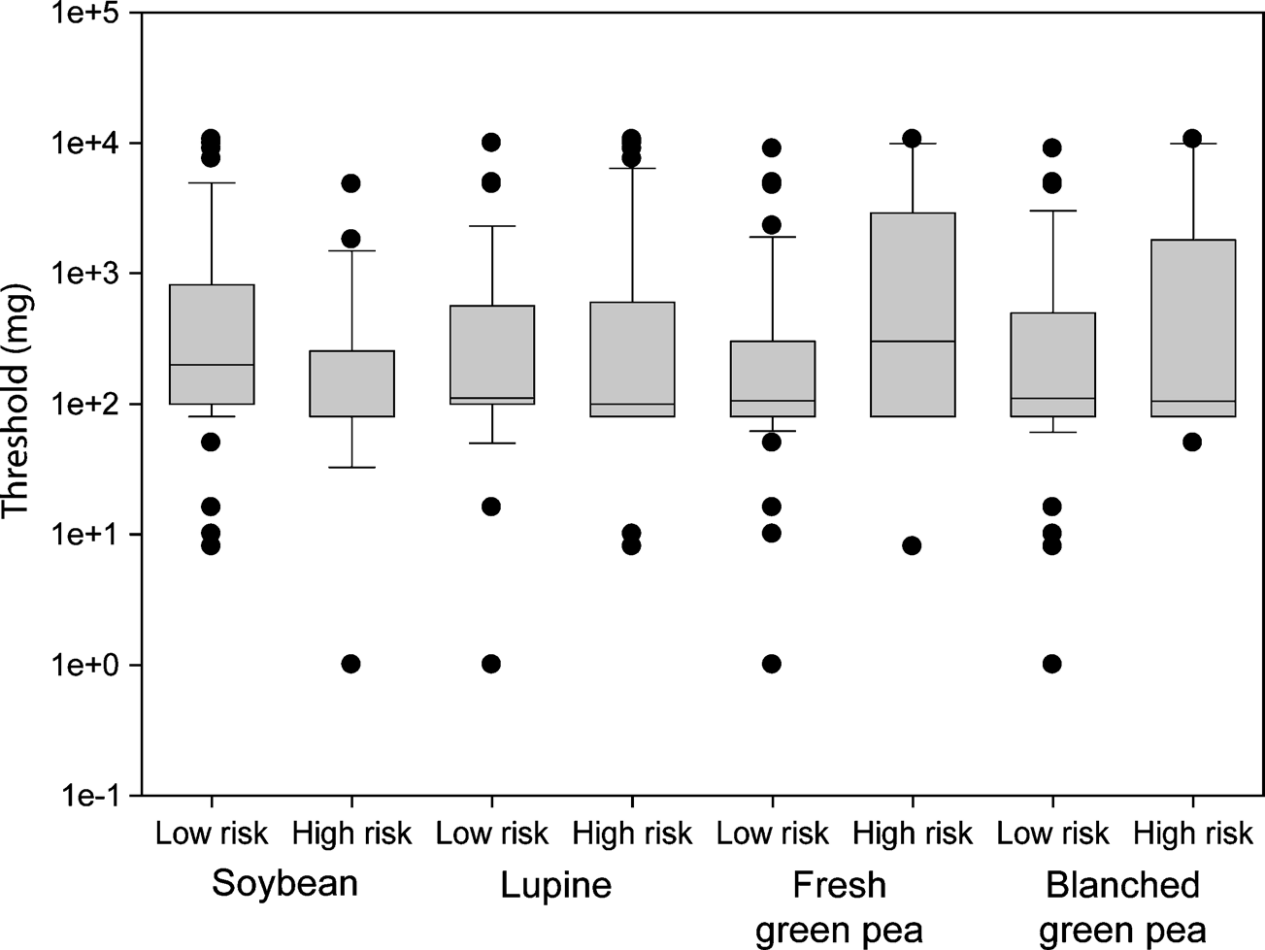
**Box plot depicting the comparison between high- and low-risk classification and threshold doses of the peanut challenge**.

The outcome of the risk assessment was also compared with the threshold of the peanut challenge (Figure 3). Interestingly, no correlation was observed for any of the legumes.

## Discussions

There is increasing evidence that patients with peanut allergy frequently cross-react serologically with other legumes, but only a subpopulation of these patients experience clinical reactions to the legumes. The frequency of clinical cross-reactivity to legumes in peanut-allergic patients is not clear because these patients either avoid legumes, are not aware of the existence of legumes in different foods, or refuse to be orally challenged with these foods.

The aim of the present study was to examine frequency of serological cross-reactivity to soybean, lupine, fresh, and blanched green pea and to compare these results with challenge and case history outcome. This approach was the basis for the development of an algorithm by which it is possible to divide the peanut-allergic patients into a high- and low-risk group reflecting the precautions the patients must take in the diet.

Histamine release as an independent diagnostic test could not predict the outcome of the algorithm giving the high- and low-risk classification. The comparison of SPT and sIgE with the algorithm outcome was not performed because SPT and sIgE were a part of the algorithm.

We expected the most sensitive peanut-allergic patients to also experience more clinically relevant cross-reactivity to the other legumes. Interestingly, the high- and low-risk classification was not dependent of the threshold dose of the peanut challenge.

Twenty-eight percent of the patients had a positive SPT to soybean, whereas for only 2 of 44 patients (5%; the 22 with a negative case history were included as challenge negative), the allergy was confirmed clinically by challenge. These numbers are in agreement with Bock and Atkins[15]who investigated 32 clinically confirmed peanut-allergic patients. They found that 10 patients (31%) had a positive soybean SPT, whereas only 1 patient (3%) had a clinical reaction after ingestion of soybean.

Case history reports from the patients and/or caretakers reflect that lupine is a new and unknown source of allergens important for peanut-allergic individuals. Only 3 patients were aware of having ingested or reacted to lupine, and of these, 1 had a positive case history. These data indicate that the awareness of lupine as a food allergen in Denmark is still sparse. A search for the combination of "peanut," "allergy," and "lupine" at PubMed gave only 13 hits, where the first is published in 1994 and is a report on reactions to lupine added to pasta [16]. It is important to emphasize that the risk of coallergy to lupine for peanut-sensitized subjects might be higher than for other legumes, but more knowledge needs to be gained on this matter. Moneret-Vautrin et al[17]investigated 24 peanut-allergic patients, and they found that 44% had a positive SPT to lupine, which is in agreement with our data where 45% had a positive lupine SPT. These authors also found that the risk of crossed peanut-lupine allergy was higher compared with cross-allergy to other legumes. This risk combined with the fact that lupine is added frequently to foods as an excellent protein source is making lupine an allergen of interest for future research.

Positive SPT frequencies were 41% and 16% for fresh and blanched green pea, respectively. Bernhisel-Broadbent and Sampson[7]investigated 69 patients with 1 or more positive SPTs to legumes in 1989 and found that 26% of pea SPTs were positive (it is not stated whether fresh or blanched green pea was used).

Eleven patients reported symptoms upon ingestion of fresh green pea, whereas no patients observed symptoms after ingestion of blanched green pea. The degradation of pea allergens by heat treatment can explain this. Peanut and lupine has been reported to be stable to heat treatment [18-20]. This stability can explain that clinically relevant reactions are seen to peanut and lupine.

Many patients were not challenged in the study but were still assigned to the high-risk group. Although no scientific reason is given for this classification, it is advisable from a practical point of view to consider these patients as high-risk patients until the opposite has been proven.

This study is the first to standardize the process of diagnosing peanut-allergic children with regard to possible cross-reactions to other foods. By using the algorithm presented in this study, it is possible to advise peanut-allergic children and caretakers on which other legumes to avoid in their diet.

## Notes

Louise Bjerremann Jensen was funded by a grant from the Faculty of Life Sciences, University of Copenhagen. The study was funded by EU Commission (FAREDAT, QLRT-2001-00301).

Presented at Danish Society of Allergology Annual Meeting, Kolding, Denmark, August 12-13 2005. European Academy of Allergology and Clinical Immunology, Gothenburg, Sweden, June 10-14 2006.

## Competing interests

The authors declare that they have no competing interests.

## Acknowledgements

The authors thank Lise Bo at RefLab ApS and Ulla Johannessen at the Allergy Centre, Odense University Hospital.
